# Novel methods for the detection of glutathione by surface-enhanced Raman scattering: A perspective review

**DOI:** 10.1016/j.heliyon.2024.e41588

**Published:** 2024-12-31

**Authors:** Mohammad Kamal Hossain, Genin Gary Huang, Mohammad Mozahar Hossain

**Affiliations:** a Interdisciplinary Research Center for Sustainable Energy Systems (IRC-SES), Research Institute, King Fahd University of Petroleum & Minerals (KFUPM), Dhahran, 31261, Kingdom of Saudi Arabia; b Department of Electrical Engineering (EE), Fahd University of Petroleum & Minerals (KFUPM), Dhahran, 31261, Kingdom of Saudi Arabia; c Department of Medicinal and Applied Chemistry, Kaohsiung Medical University, Kaohsiung, 807, Taiwan; d Department of Chemical Engineering, Fahd University of Petroleum & Minerals (KFUPM), Dhahran, 31261, Kingdom of Saudi Arabia; e Interdisciplinary Research Center for Refining & Advanced Chemicals (IRC-RAC), Research Institute, King Fahd University of Petroleum & Minerals (KFUPM), Dhahran, 31261, Kingdom of Saudi Arabia

**Keywords:** Surface-enhanced Raman scattering, Glutathione, Silver nanoparticles, Heat-induced method, Reversed reporting agent method

## Abstract

Detection of biomolecules, Glutathione (GSH) in particular, is important because it helps assess antioxidant capacity, cellular protection, detoxification processes, and potential disease associations. Monitoring glutathione levels can provide valuable information about overall health and well-being. Many medical disorders have been connected to glutathione levels. Higher glutathione levels have been seen in several cancer cell types, which may increase their resistance to radiation and chemotherapy. Glutathione levels can be measured through various methods, such as colorimetric assays and fluorescent probes. However, surface-enhanced Raman scattering (SERS) has been known as an efficient and selective technique for biomolecule detection. Here in this perspective review, we have reported two distinctive methods based on SERS technique in detection of GSH; heat-induced method and reversed reporting agent method. Several variables that can impact the detection scheme were elaborated in the "heat-induced method," including pretreatment, nanoparticle reduction time, the process temperature, the pH of the colloidal solution, the concentration of citrate buffer, and the concentration of participating nanoparticles. To choose the best reporting agent for a reverse reporting scheme using SERS approaches, several reporting agents were examined in the second method. In order to grasp the situation at hand, biomolecule detection—specifically, GSH detection schemes—was briefly discussed. SERS spectroscopy and its associated terminology were then covered followed by the perspective and outlook of GSH detection at the end. To meet the demands of real-time applications in everyday life and to enhance SERS methods for biomolecule detection—in particular, GSH detection—such a thorough investigation is unavoidable.

## 1 Introduction

The detection of biomolecules plays a crucial role in various fields, ranging from healthcare and diagnostics to environmental monitoring and food safety [[1], [2], [3]]. Biomolecule detection methods are used to detect diseases, allergies, and pollutants in food items to some extent [2,[4], [5], [6]]. Through the process, healthcare practitioners track the stages of illnesses, make early diagnoses, and predict treatment decisions [2,4,7]. Researchers utilize detection techniques to find possible therapeutic targets, assess the effectiveness of treatment, and investigate how certain biomolecules interact with patients [8,9]. Biomolecule identification is essential for evaluating the effects of poisons, infections, and pollutants in environmental monitoring [1]. The presence of hazardous compounds can be identified and determined by their concentration, and thus appropriate action can be taken to safeguard human health and the environment. Biomolecule detection is an essential process in biotechnology that supports the manufacturing and quality assurance of enzymes, biotherapeutics, and other bioproducts [5]. Through precise biomolecule detection, scientists can guarantee the efficacy and minimize hazards associated with these goods by assuring their potency, purity, and safety. In this context, the detection of glutathione (GSH) at an early stage is of vital importance.

GSH plays a critical function in preserving cellular health and guarding against oxidative stress [10,11]. GSH is an important antioxidant that aids in shielding cells from free radical damage [11]. Researchers and medical experts evaluate the antioxidant capability of a cell and investigate any abnormalities that could be linked to a number of disorders by quantifying GSH levels. An increased risk of heart disease, asthma, neurological illnesses, and several forms of cancer in their early stages has been linked to GSH deficiency [[12], [13], [14], [15]]. Abnormal GSH levels are also linked to a number of clinical conditions, such as inflammatory diseases, Parkinson's disease, diabetes, liver damage, Alzheimer's disease, and cardiovascular diseases [[16], [17], [18]]. Thus, the development of sensitive and focused GSH detection tools is imperative.

### 1.1 Raman scattering technique

The phenomenon known as Raman scattering, named after the Indian scientist Sir C.V. Raman who first observed it in 1928, provides insight into the vibrational modes and molecular structure of a variety of materials [19,[20], [21]]. Light scattered by materials or molecules in an inelastic manner is the basic process of Raman scattering. When interacting with a substance, the bulk of photons in a monochromatic light beam simply change their path without altering the energy and the phenomenon is known as elastic scattering. However, a very small portion of the photons undergo a wavelength and frequency shift as a result of their energy exchange with the material or molecule and the phenomenon is known as Raman scattering. The Raman scattering process can be better understood in terms of the connection between molecule vibrational modes and light yielding stimulated vibrational and rotational states of a molecule. In terms of energy state, there are two possibilities in the Raman effect, known as Stokes scattering and anti-Stokes scattering. Stokes scattering is the process where the scattered photon has less energy and a longer wavelength than the initial photon as shown in Fig. 2a. On the other hand, anti-Stokes scattering occurs when the photon that is dispersed has a higher energy and a shorter wavelength than the initial photon.

The mechanism of Raman scattering is inelastic, meaning that incoming photons either acquire or lose energy according to the vibrational and rotational motion of analyte as mentioned above. The resultant Raman spectra give chemical "fingerprints" for the identification of the analyte because they contain bands that correspond to vibrational or rotational transitions unique to the molecular structure. This is a weak event, though, as only about 1 in 10^6^–10^10^ photons are dispersed in an inelastic manner [22]. Raman scattering cross-sections typically range from 10^−29^ cm^2^/molecule. On the other hand, the cross-sections of standard fluorescent dyes are ∼10^−15^ cm^2^/molecule. Note that the cross-section may be significantly increased via resonant Raman scattering. For instance, rhodamine 6G (R6G) at incident excitation of 532 nm can have a resonant Raman cross-section as high as 10^−23^ cm^2^/molecule. However, the resonant Raman cross-section cannot compete with the fluorescent cross-section of a standard dye.

### 1.2 Glutathione and its effect

**Fig. 1:**
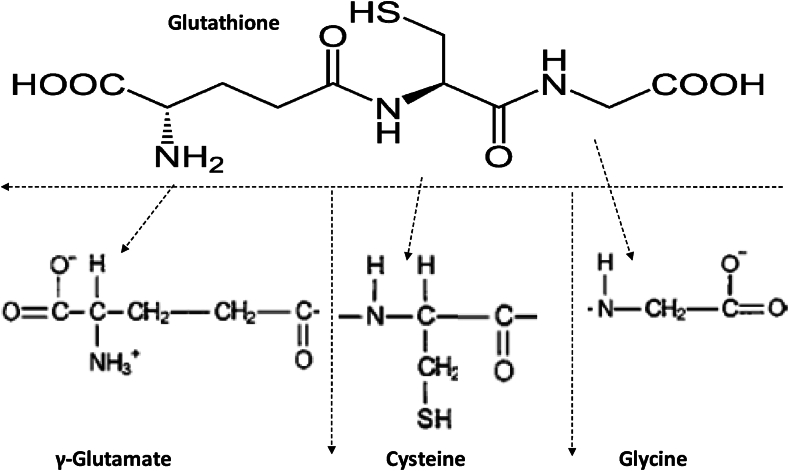
Molecular structure of GSH and constituent peptides; (a) y-Glutamic acid (glu), (b) Cysteine (cys) and (c) glycine (gly).

Known as the "master antioxidant," GSH is an essential chemical that exists in all of our cells [10,11]. It is essential to preserving our general health and wellbeing. As seen in Fig. 1, GSH is fundamentally a tripeptide made up of three amino acids: cysteine, glycine, and glutamic acid. Its main job is to shield our cells from oxidative stress and free radical damage by acting as a potent antioxidant [10,11]. Unstable chemicals known as free radicals may harm lipids, proteins, and DNA, which can result in a number of health problems. The ability of GSH to donate an electron to neutralize these dangerous free radicals is one of its primary functions. By transforming the free radicals into stable molecules, this mechanism stops them from doing more harm. As mentioned earlier, GSH is necessary for the detoxification processes of the human body. The liver needs GSH in order to carry out detoxification effectively and thus maintain a balanced and healthy internal environment. Additionally, GSH supports the enhanced function of immune cells, including T cells and natural killer cells, which are essential for defending the body against illnesses and infections [[12], [13], [14], [15]]. It is crucial to understand that a variety of factors influence the GSH levels of the human body, including but not limited to aging, chronic stress, a poor diet, environmental contaminants, and certain medical conditions.

**Fig. 2:**
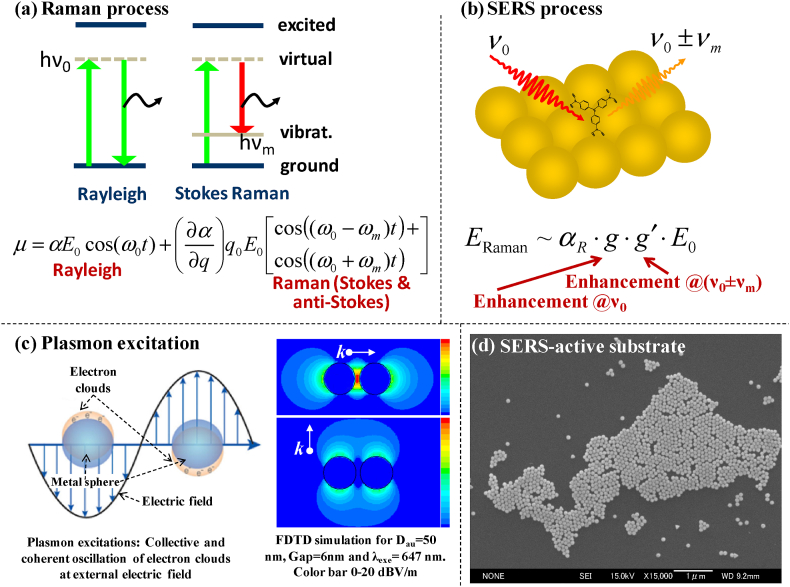
(a) Band energy diagram of Raman process wherein the electrons excited from ground state return to vibrational level via virtual state yielding a shift in incident energy, (b) Simplified schematic of SERS process along with the formulation of two-fold enhancements; one, due to incident field and two, due to scattered field, (c) Polarized electrons cloud in metal sphere upon laser excitation, and finite-different time-domain analysis of a metal NP dimer. Localized EM field distribution occurs only in s-polarization. Color bar represents 0–20 dBV/m and (d) SEM micrograph of a typical SERS-active nanoparticulate consisted of 60 nm Au NPs on glass substrate.

### 1.3 Detection schemes of glutathione

There are several detection schemes of GSH reported so far such as electrochemical analysis, high-performance liquid chromatography, colorimetric assays, and fluorescence spectrometry [[22], [23], [24], [25]]. Although these techniques work well for detection of GSH, there are constraints related to expensive equipment, challenging sample preparation, and limited ability to prevent interference [26,27]. Monochlorobimane and ThioStar are examples of fluorescent probes that bind to GSH specifically and create stable fluorescent adducts providing strong fluorescence intensity of GSH [28]. Colorimetric assays make use of chemical processes that result in a color shift corresponding to GSH level. GSH levels can be determined by using spectrophotometric measurement of the color shift. On the contrary, mass spectrometry is capable of detecting GSH with extreme precision and sensitivity [29]. Lately, genetically encoded nanosensors are being utilized to detect GSH levels in live cells producing a fluorescence resonance energy transfer (FRET) signal [30]. Over the past ten years, surface-enhanced Raman scattering (SERS) spectroscopy has received a lot of attention in biological and medical detection applications because plasmonic nanostructures combine high sensitivity with molecular Raman spectra [[31], [32], [33], [34], [35]]. It is well known that Raman scattering signals are significantly increased when target molecules are placed at or very close to the metallic nanostructure (often Au and Ag). The widely accepted reason for the enhancement is that the surface plasmon resonance of the metallic nanostructure, driven by a laser, boosts the local electromagnetic fields (EM fields), hence improving the Raman signal of the surrounding probe molecules. Larsson and Lindgren recorded the tripeptide GSH spectra using chromatographic beads containing gold (Au) nanoparticles [36]. However, the detection of GSH through the SERS technique was found insensitive. Following that, a "heat-induced SERS sensing method" was developed by Ozaki and colleagues for the quick measurement of GSH in aqueous solutions [37]. It is well-acknowledged that even with the low detection limit attained, interference was unavoidable. To the extent, surface-enhanced competitive Raman spectroscopy was developed to quantify GSH [38]. In these techniques, the additional GSH would take the place of a Raman probe on the surface of SERS-active substrate which would reduce the signal. The Raman probe mediated competitive technique was useful for GSH detection, but the catastrophic lack of simple aggregation plagues the usage of sol-gel-based SERS substrates in the works.

In this perspective review, we have elaborated two distinguished methods, called hereby “heat-induced method” and “reversed reporting agent method” for the detection of GSH by SERS. In the “heat-induced method”, several factors that can affect the detection scheme such as pretreatment, reduction time in nanoparticles (NPs) synthesis, temperature used in the heating process, pH of the colloidal solution, concentration of citrate ligands and concentration of participating NPs were elaborated. In the second method, several reporting agents were checked to choose the suitable one for such a reverse reporting scheme in SERS techniques. Biomolecule detection, particularly GSH detection schemes, was highlighted in brief followed by SERS spectroscopy and its related terminologies to understand the scenario in the beginning. Finally, the perspective and outlook of GSH detection have been elaborated. Such an extensive study is inevitable to improve the SERS techniques for biomolecule detection, GSH detection in particular, as well as to face the challenges for real-time applications in daily life.

## 2 Surface-enhanced Raman scattering

SERS is a specific type of spectroscopy that falls within the category of Raman spectroscopy [19,20,39]. SERS is the study of specific molecules adsorbed on a metal surface that has been carefully prepared using an intense Raman spectrum often with an enhancement factor of 10^8^-10^10^. Fig. 2a displays a simplified schematic depicting the Raman process. A magical location, referred to as the "active site" or "hot-site," arises at the intersections or close to NPs when exposed to incident excitation. The behavior is consistent with the dispersion of strong electromagnetic (EM) fields mediated by localized surface plasmon resonances (LSPR). The electron cloud distribution of typical NPs and coherent oscillation caused by incoming light are shown in Fig. 2b.

The distribution of the electromagnetic field around the NPs may be readily obtained by applying the time-variant Maxwell equation. Such field distributions in both s- and p-polarization are shown in Fig. 2c [40,41]. As a result, the analyte near the center of this distribution will have the most impact and exhibit the largest SERS enhancement. SERS is a powerful spectroscopic technique that enhances the Raman scattering signal of molecules adsorbed on nanostructured metal surfaces or nanostructures. It facilitates the detection and characterization of molecules with high sensitivity and selectivity, even at low concentrations. The principle behind SERS lies in the interaction between the incident light and the metal surface. When a molecule is adsorbed onto a rough or nanostructured metal surface, such as Au or silver (Ag), the localized surface plasmons of the metal interact with the incident light, leading to an enhancement of the Raman scattering signal. This enhancement can be several orders of magnitude higher compared to conventional Raman spectroscopy. Two groups, the Van Duyne group and the Creighton group, independently verified the effect of a roughened surface on increased Raman scattering in 1977, although the first observation ever by Fleischmann et al. was reported in 1974 [19,20,[42], [43], [44]]. The EM effect was postulated by R. Brust and VanDuyne, whereas the charge-transfer (CT) effect was proposed by Albrecht and Creighton [43,[45], [46], [47]]. In 1997, two distinct groups using various experimental settings reported on the astounding single molecule detection capabilities of SERS. Following the year, many other reputed groups, such as Moskovits et al., Zhang et al., and Xu et al., have also revealed the specifics of the underlying theory [[48], [49], [50]].

### 2.1 Plasmon excitation: Localize surface plasmon resonance and coupling

It is well-acknowledged that metal NPs are made up of positive ions encircled by a "sea of electrons". This "sea of electrons" is incoherent while in motion and can move and oscillate coherently when stimulated by electromagnetic radiation. This can create powerful EM fields and generate plasmons, which are shown in Fig. 2c. Therefore, when an incident photon induces surface plasmon resonances into their resonant mode, the local electromagnetic field surrounding the metal gets enhanced significantly [19,20,40,41]. Subsequently, incident photons and surface plasmons combine to generate surface plasmon polariton, a wave-like energy that travels along the surface. However, surface plasmon mode cannot couple directly to free-space electromagnetic radiation of the same energy because they travel too slowly and the corresponding wavevector is too large to satisfy momentum and energy conservation requirements. Fortunately, at the low k-vector limit, these surface modes combine with the free electromagnetic field to generate a polariton-type excitation.

The resonant frequency of LSPR strongly depends on several factors, such as the composition, size, geometry, dielectric environment, and interparticle gaps of the constituent NPs [41]. The interaction between the incident photon and the NPs leads to the unique characteristics in absorption and scattering of light, resulting in unique optical properties. One of the key features in this context is the enhancement of the electric field near the surface of the NPs. This enhanced electric field can be utilized for various applications, such as surface-enhanced spectroscopy techniques like SERS and surface-enhanced fluorescence. The enhanced electric field near the NPs can significantly amplify the Raman scattering signal of molecules adsorbed on their surface, enabling highly sensitive molecular detection. LSPRs coupling refers to the interaction and influence between localized surface plasmon resonances (LSPRs) of neighboring NPs or nanostructures [51,52]. When NPs are in close proximity to each other, their LSPRs can couple, leading to changes in the optical properties and behavior of the system. LSPR coupling can lead to an enhanced electric field between the NPs. LSPR coupling can result in the hybridization of plasmonic modes between neighboring NPs. This leads to the formation of new collective plasmon modes with distinct properties compared to the individual LSPRs. The hybridization can affect the intensity, polarization, and spatial distribution of the plasmonic modes, offering new opportunities for tailoring the optical response of plasmonic systems.

### 2.2 SERS mechanisms: Electromagnetic enhancement and chemical enhancement

According to the SERS process as explained in Fig. 2a, any enhancement in Raman scattering intensity must originate from either an enhancement of α (molecular polarizability) or *E* (the electric field caused by the incoming radiation), as the intensity is directly proportional to the square of the induced dipole moment (*P*), whereas $\bar{P}=\alpha \cdot \bar{E}$. Chemical enhancement refers to the enhancement connected with α, whereas EM enhancement is the one linked with E.

**Fig. 3:**
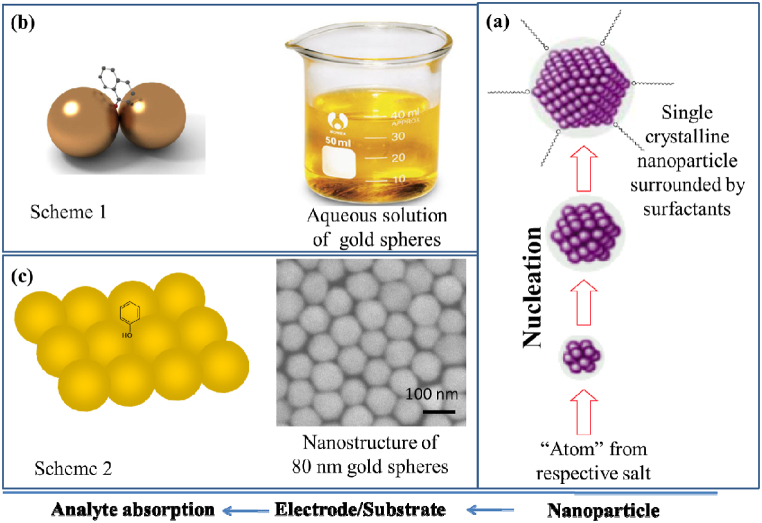
Schematic presentation showing (a) the mechanism of forming NPs in bottom up process, (b) SERS-active colloids and (c) SERS-active nanoparticulate derived from colloidal solution.

The two-fold EM field enhancement is thought to be the primary cause of the large enhancement factor, which is on the order of 10^8^‍-10^10^. Fig. 3 shows this two-fold amplification of the EM field. To summarize, incoming photon energy that is irradiated on an adsorbent is scattered from an adsorbate (shown by M_1_), and the scattered photon from the molecules resonantly combines with the plasmon, leading to further amplification (represented by M_2_) [39]. Strong Raman scattering light is therefore released. The SERS enhancement factor, M, is provided by,

$$M={\left|\frac{E^{Loc}\left(\lambda_{L}\right)}{E^{I}\left(\lambda_{L}\right)}\right|}^{2}\times {\left|\frac{E^{Loc}\left(\lambda_{L}\pm \lambda_{R}\right)}{E^{I}\left(\lambda_{L}\pm \lambda_{R}\right)}\right|}^{2}=M_{1}\left(\lambda_{L}\right)\times M_{2}\left(\lambda_{L}\pm \lambda_{R}\right)$$

where λ_L_ is the excitation wavelength, +λ_R_ and –λ_R_ are the wavelengths of the anti-Stokes and Stokes Raman shifts, respectively, and M_1_ and M_2_ are the first and second enhancement factors. The amplitudes of the incident and local electronic fields are represented by E_I_ and E_Loc_, respectively.

There are several predictions suggesting that the abovementioned two distinct enhancement processes could be going on simultaneously. Through a specific interaction with the surface of a NP, an analyte can transfer charge from occupied surface levels to the analyte or from the analyte into unoccupied levels on the nanoparticle's surface. While it is true that the SERS signal enhances significantly (10–1000) when the incident or Raman-scattered photons become nearly resonant with the charge transfer excitation of the metal-analyte system, experimental results have led to a general acceptance of the order of 10^6^ to 10^8^ solely due to the EM mechanism [19,20,40].

### 2.3 SERS-active substrates: Colloidal and nanoparticulate

Uncertain topology of SERS-active substrates due to differences in surface roughness that impact SPR excitations, poor colloidal size distribution, and challenging strategies for producing nanostructured substrates, qualitative research of SERS has suffered up to this point. Different strategies have been devised to create consistent and repeatable LSPRs-active nanostructures from a variety of materials. The wet chemical route is the most straightforward and practical method to date for producing Au/Ag NPs. Although sophisticated and state-of-art techniques do exist, the most common way for creating these NPs is the reduction of the corresponding salt. On the other hand, nanostructures fabricated from colloidal metal particles are highly distinctive and attract a lot of interest.

For SERS applications, a variety of SERS-active substrates have been created [[53], [54], [55], [56], [57], [58], [59], [60]]. Metallic NPs, such as Au and Ag NPs, are frequently employed as substrates for SERS. To get distinct plasmonic characteristics and amplify the Raman signal of adsorbed molecules, these NPs may be synthesized in a range of sizes and shapes, such as spheres, rods, and triangles as shown in Fig. 3a. A colloidal solution of NPs can make efficient interstitials and the Raman signal of a target analyte at the junction of such NPs conjugate can be enhanced as shown in Fig. 3b. On the other hand, enhanced SERS activity can be obtained from nanostructured surfaces including patterned or rough characteristics as shown in Fig. 3c. In order to create features that enhance the electromagnetic field close to the surface and contribute to the SERS signal amplification, these surfaces can be designed by methods such as electron beam lithography, nanoimprinting, or laser ablation. Noble metal NPs like Au or Ag NPs that are ordered or nanoparticulate have been investigated as extremely SERS-active surfaces. These arrays can offer large amplification factors for Raman scattering and show strong plasmonic coupling effects. SERS-active substrates can also be fabricated by combining different materials, such as metal-dielectric hybrids or metal-semiconductor hybrids. These hybrid structures can provide additional enhancement mechanisms and enable tailored SERS responses for specific applications.

## 3 Heat-induced method for glutathione detection

**Fig. 4:**
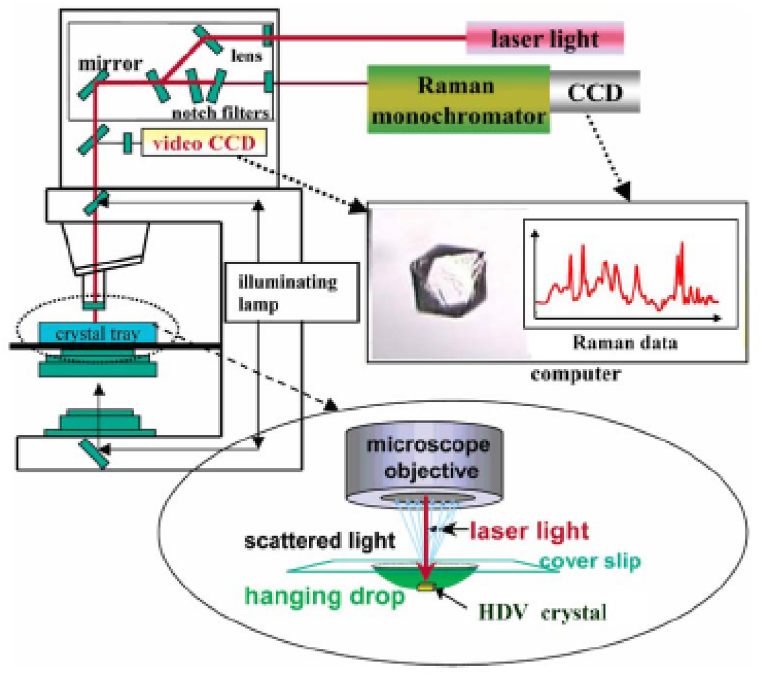
A freehand schematic of far-field experimental setup adopted in the proposed heat-induced method.

A modified Lee and Meisel technique was employed to create the citrate-reduced Ag colloidal solution used in this investigation. In brief, 90 mg of Ag nitrate and 500 mL of triply distilled water

were combined, and the precursor was rapidly refluxed to a boil while being violently agitated. Ten milliliters of 1 % (w/v) sodium citrate aqueous solution was added as soon as the mixture began to boil, and it was left to boil for the whole set heating time. The reduced Ag colloidal solution was removed from a hot plate and immediately placed in a cold bath to halt the reduction of Ag colloids further. All SERS and Raman spectra were recorded using the HoloProbe VPT system (Kaiser Optical Systems, Ann Arbor, MI, USA) as shown in Fig. 4. The analytes incubated Ag colloidal solution was transferred using an aluminum pan (0219-0062, PerkinElmer). Throughout the whole experiment, unless otherwise specified, 50 mL of Ag colloidal solution was mixed with 50 mL of the 10 mM citrate buffer solution (pH = 4.0) before the SERS measurement. The excitation source was set to 785 nm line of NIR laser (InvictusTM; Kaiser Optical Systems) with a spot size of 10 μm. At the sample location, the laser power was around 15 mW, and the exposure duration was adjusted to 1 s with 10 accumulations for each SERS measurement. Since nanoscale morphology has been known to be crucial for SERS measurement, Ag colloidal aggregates after different pretreatments were investigated using field emission scanning electron microscopy (FESEM, JSM-6700F, JOEL) and have been elaborated in the later part of the text.

### 3.1 Role of pretreatment on the detection

**Fig. 5:**
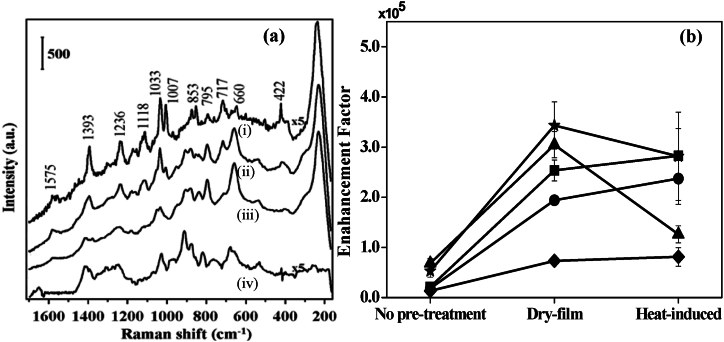
(a) SERS spectra of GSH; insets (i)-(iv): 10 μM GSH solution without any pretreatment, by dry-film method, and by heat-induced method respectively and inset (iv) Raman spectrum of 0.5 M GSH aqueous solution as reference. Vertical bar represents the intensity scale and (b) SERS Enhancement factors of C-S stretching (■), -COO- bending (●), C-C stretching of C-COO- (◆), C-N stretching of glycine terminal (▲), and C-N stretching of glutamic acid terminal (★) [61].

The EM mechanism is dominating in SERS techniques and it is important to make the constituent colloids active to form more active sites or hot sites. Several experimental conditions, such as natural dry (hereby called the ‘dry film method’) and external heat (hereby called the ‘heat-induced method’) were applied for this purpose. The GSH incubated Ag colloids were dried using the dry film method for 90 min at room temperature and the heat-induced method for 3 min at 100 °C. Fig. 5a shows the SERS spectra of 10 μM GSH solution. The specimens without any pretreatment and those prepared by dry-film method and by heat-induced technique are shown in insets (i)-(iii) of Fig. 5a respectively. Referencing the Raman spectra, Fig. 5 (iv) displays an aqueous solution of 0.5 M GSH. As shown in the inset of Fig. 5a, the SERS peaks were noted distinctively for GSH solution without any pretreatment. Although it took a while to prepare the specimen, the dry-film approach provided a higher enhancement factor with reference to that recorded for GSH solution without any pretreatment. However, when the heating method was used, the SERS intensities of GSH solution were found to be higher compared to those obtained for specimens prepared by the dry-film method as shown in Fig. 5b. From FESEM micrographs shown in the later part of the text, it has been clear that treatments do have influence in forming more active sites or hot sites. Table 1 displays the principal peaks identified and their corresponding band assignments in the GSH SERS and Raman analyses. Even at temperatures below 100 °C, it is seen that the majority of the GSH bands either stay in place or very slightly move during the drying process.

**Table 1:** Raman bands and their assignments [62] in the SERS spectra of 10 μM GSH solution incubated Ag colloids using various techniques, as well as those in the Raman spectrum of 0.5 M GSH aqueous solution.

Raman	SERS			Assignments
0.5 M aqueous solution	No pretreatment	Dry-film	Heat-induced SERS sensing method	
2571				S-H stretching
1665				Amide I
1417	1393	1393	1414	-COO^-^ symmetric stretching
1265	1234	1235	1258	Amide III
	1115	1115	1125	N-C stretching + C-C stretching
		1053	1053	C-N stretching of Glu
1031	1034	1034	1034	C-N stretching of Gly
	1007	1007	1007	C-C stretching
		905	905	C-COO^-^ stretching
880	875	880	880	C-C stretching
819	853	838	838	C-CN stretching
	795	795	795	-COO^-^ bending
765	717	717	717	-COO^-^ deformation
678	655	660	660	C-S stretching
536		539	539	N-C-C deformation
	236	231	231	Ag-S stretching

**Fig. 6:**
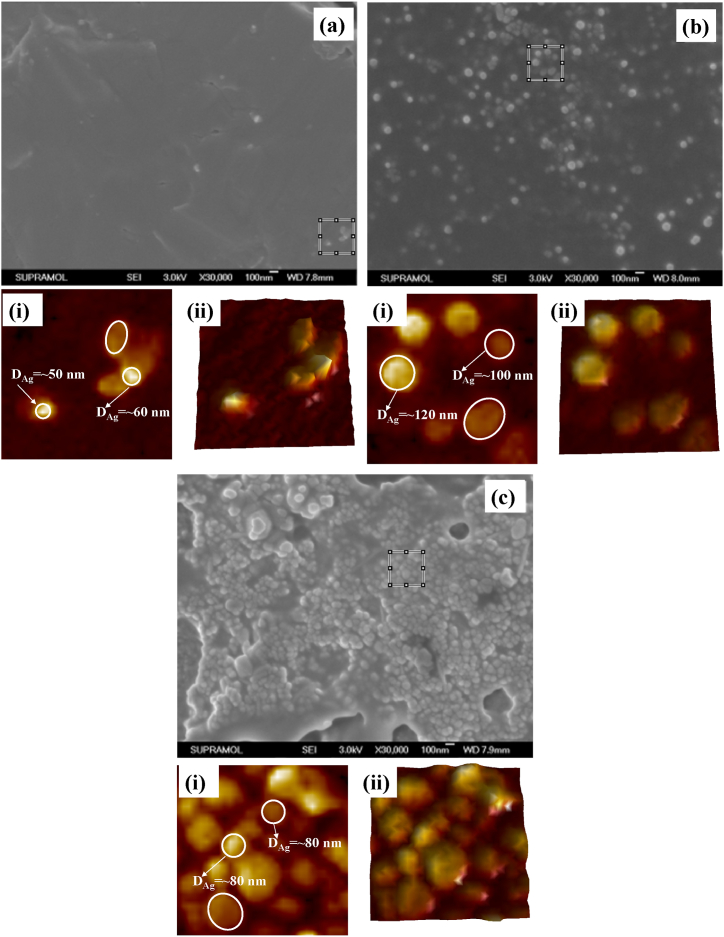
FESEM micrographs GSH incubated Ag colloids (a) without any pretreatment; insets (i)-(ii): zoom-in view and corresponding hawk-eye view respectively of a small area (500 nm × 500 nm) as marked by the white square therein, (b) with dry-film method; insets (i)-(ii): zoom-in view and corresponding hawk-eye view respectively of a small area (500 nm × 500 nm) as marked by the white square therein, and (c) and with heat-induced method; insets (i)-(ii): zoom-in view and corresponding hawk-eye view respectively of a small area (500 nm × 500 nm) as marked by the white square therein [37].

The FESEM images of Ag colloids before and after various pretreatments are displayed in Fig. 6. There were very few Ag NPs visible in the absence of any preparation as shown in Fig. 6a. The insets (i) and (ii) of Fig. 6a represent zoom-in view and corresponding hawk-eye view, respectively, of a small area (500 nm × 500 nm), as marked by the white square therein. It was noted that most of the particles were in the range of 50–60 nm of diameter, although the shapes were found to be not uniform. However, using the dry-film approach, partial aggregations of the Ag NPs were formed as shown in Fig. 6b. The insets (i) and (ii) of Fig. 6b display zoom-in view and corresponding hawk-eye view respectively of a small area (500 nm × 500 nm) as marked by the white square therein. It was noted that most of the particles were found uniform along with the diameter of the NPs in the range of 100–120 nm. Conversely, the heat-induced approach caused the Ag NPs to aggregate more, which produced more active sites as shown in Fig. 6c. The insets (i) and (ii) of Fig. 6c depict zoom-in view and corresponding hawk-eye view respectively of a small area (500 nm × 500 nm) as marked by the white square therein. It was noted that most of the particles were found uniform along with a higher density of interparticle junctions. Therefore, it was speculated that the heat-induced approach should have a considerably larger enhancement factor than other treatment methods because of these notable disparities between the Ag NPs with different treatments. The SERS measurements as shown in Fig. 5 indeed verified the speculation.

### 3.2 Role of reduction time on the detection

**Fig. 7:**
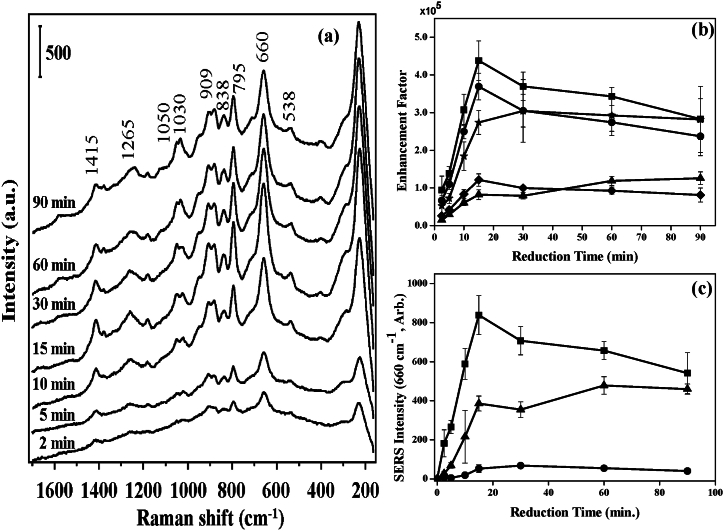
(a) SERS spectra of 10 μM GSH solution prepared by heat-induced method for different reduction times of Ag colloids. Vertical bar represents the intensity scale. (b) SERS Enhancement factors of C-S stretching (■), -COO- bending (●), C-C stretching of C-COO- (◆), C-N stretching of glycine terminal (▲), and C-N stretching of glutamic acid terminal (★) at different reduction times. (c) SERS intensity of 10 μM GSH solution at 660 cm^−1^ band (i.e. C-S stretching) with heat-induced method (■), with dry-film method (▲), and without pretreatment (●) for different reduction times [61].

The enhancement of GSH detection was found to depend on reduction time during the synthesis of Ag colloids. It is well-known that nucleation as well as the ultimate size of the colloids relies on the reduction process in synthesis and thus it influences the SERS enhancement. Here, GSH was detected using Ag colloids prepared with varying reduction periods and pretreatments as shown in Fig. 7. The SERS band, which is positioned at 660 cm^−1^ corresponding to C-S stretching mode, was used as a reference to define SERs enhancement factors under this investigation. Without any pretreatment, it was shown that the SERS intensity is low, while the heat-induced approach produced the greatest boost as shown in Fig. 7c. With longer reduction times for the dry-film approach, the SERS intensities of GSH solution were found to be higher with reference to those recorded at the specimen without treatment as shown in Fig. 7a and b. However, when the heat-induced method was applied, the SERS intensity increased rapidly and the SERS intensity reached its maximum value at around 15 min, then started to decrease a bit afterword.

### 3.3 Role of temperature on the detection

**Fig. 8:**
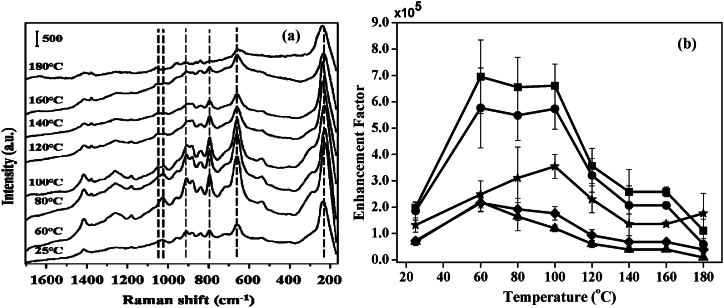
(a) SERS spectra of GSH (10 μM) by heat-induced method for different drying temperatures. Vertical bar represents the intensity scale. (b) Enhancement factors of C-S stretching (■), -COO- bending (●), C-C stretching of C-COO- (◆), C-N stretching of glycine terminal (▲), and C-N stretching of glutamic acid terminal (★) calculated from the SERS spectra obtained by heat-induced method for different drying temperatures [61].

The detection of GSH was found to depend on the drying temperature of the sample preparation. In fact, drying temperature defines how quickly the aggregation will be formed and thus influences the nature of the aggregations as well as the stability of molecules adsorbed on the surface. Herein the sample was dried by heating at the temperature from 60 °C to 180 °C prior to SERS detection as shown in Fig. 8a. Intense SERS signal was observed only in between 60 °C and 100 °C as shown in Fig. 8b. The enhancement was found low at a drying temperature of 60 °C keeping nearly stable and higher enhancement till 100 °C and decreasing the enhancement again at a drying temperature after 100 °C as shown in Fig. 8b.

### 3.4 Role of pH on the detection

**Fig. 9:**
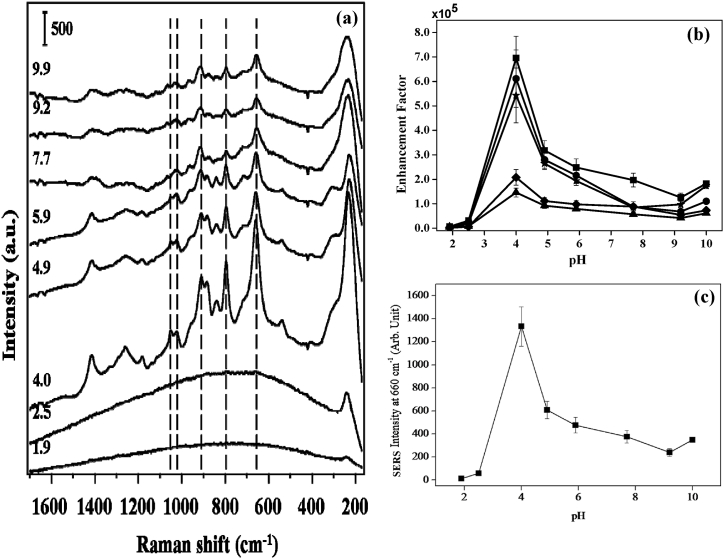
(a) SERS spectra of GSH (10 μM) by heat-induced method for different pH of Ag colloidal solutions. Vertical bar represents the intensity scale. (b) Calculated enhancement factors of C-S stretching (■), -COO- bending (●), C-C stretching of C-COO- (◆), C-N stretching of glycine terminal (▲), and C-N stretching of glutamic acid terminal (★) at different pH. (c) SERS intensity of GSH (10 μM) at 660 cm^−1^ band (i.e. C-S stretching) by heat-induced method for different pH of Ag colloidal solutions [61].

Fig. 9 shows a plot of the GSH SERS intensity measured at various pHs. Prior to adding GSH solution, HCl or NaOH solution was added to the Ag colloidal solutions to alter their pH. When the pH of the Ag colloidal solution is around 4.0, the greatest GSH enhancement factor is achieved as shown in Fig. 9a and b. The negatively charged Ag particles utilized in this investigation make it simpler for the positively charged and zwitterionic forms of GSH (pH less than 8) to be adsorbed on the Ag surfaces. Nevertheless, in extremely acidic environments, Ag aggregates may be destroyed, meaning that at pH values below 2.5, the GSH SERS signal is almost nonexistent. The other explanation is that at pH values of about 4.0, GSH induces the aggregation of Ag colloids, but at pH values of neutral or higher, no aggregation would take place. The SERS band, which is positioned at 660 cm^−1^ corresponding to C-S stretching mode, was used as a reference to define SERs enhancement factors under this investigation as shown in Fig. 9c.

### 3.5 Role of citrate ligand on the detection

**Fig. 10:**
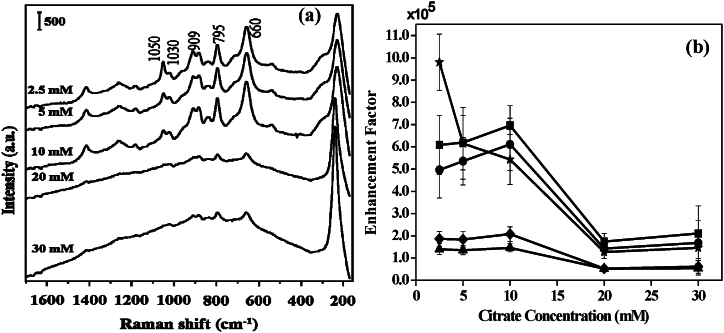
(a) SERS spectra of GSH (10 μM) by heat-induced method for different citrate buffer solution of Ag colloids. Vertical bar represents the intensity scale. (b) Enhancement factors of C-S stretching (■), -COO- bending (●), C-C stretching of C-COO- (◆), C-N stretching of glycine terminal (▲), and C-N stretching of glutamic acid terminal (★) calculated from the SERS spectra obtained by heat-induced method for different buffer solution of Ag colloids [61].

Using a combination of Ag colloidal solutions and citrate buffer solutions at different concentrations, SERS spectra were analyzed to confirm that the recorded SERS spectra are caused by GSH adsorption. Since the surface charges of these particles change in response to the concentration of citrate buffer solutions, if the adsorption of GSH by Ag colloidal particles influences the SERS spectra, then the GSH SERS signal would also be affected. Usually citrates are present as surfactants on the surface of Ag NPs and hence the adsorption of GSH molecules depends on the concentration of these surfactant ligands. At the same time, aggregation of colloidal NPs relies on such surfactant concentration. Fig. 10a shows the SERS intensity of GSH at different citrate concentrations. It has been shown that at citrate concentrations below 10 mM, the SERS intensity of GSH intensity is comparatively greater. The SERS enhancement factor for different bands of GSH was shown in Fig. 10b. The observation was consistent with the theory that GSH adsorption on the Ag surfaces was the source of the strong SERS signal. GSH molecules were unable to readily adsorb on the surface of Ag NPs or produce aggregation at higher citrate concentrations. However, at lower citrate buffer concentrations, GSH bonded to the Ag surface more readily, causing aggregation that intensifies the SERS signal. Further confirmation was revealed by FESEM micrographs as shown in the later part of the text.

**Fig. 11:**
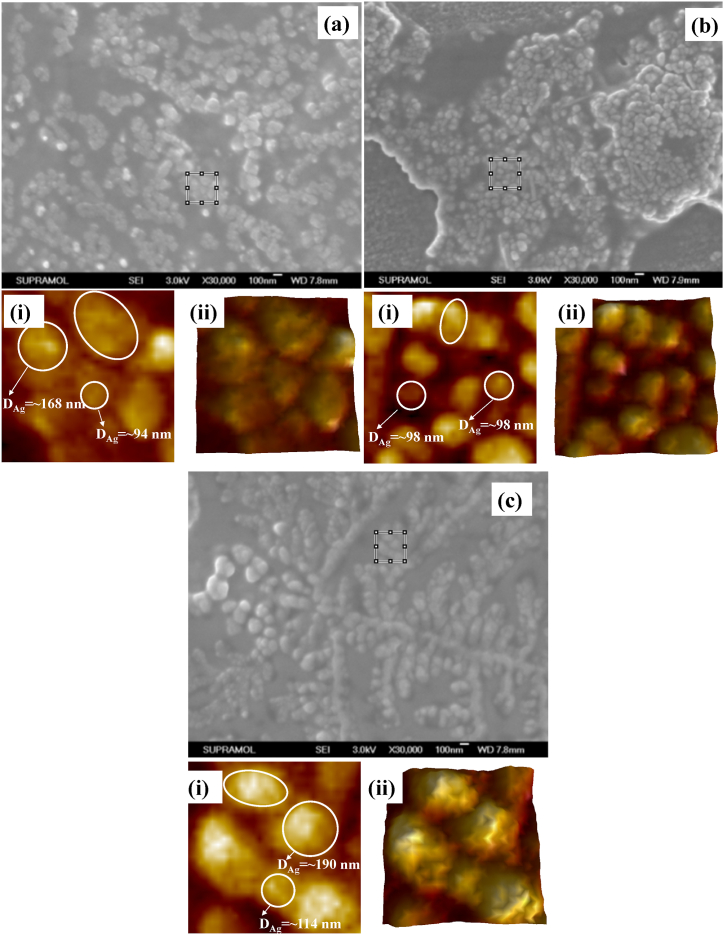
FESEM micrographs of GSH incubated Ag colloidal solutions obtained with (a) 2.5 mM; insets (i)-(ii): zoom-in view and corresponding hawk-eye view respectively of a small area (500 nm × 500 nm) as marked by the white square therein, (b), 10 mM; insets (i)-(ii): zoom-in view and corresponding hawk-eye view respectively of a small area (500 nm × 500 nm) as marked by the white square therein, and (c) 20 mM of citrate buffer solutions; insets (i)-(ii): zoom-in view and corresponding hawk-eye view respectively of a small area (500 nm × 500 nm) as marked by the white square therein. Silver colloids with the reduction time of 15 min were used, the GSH concentration was 10 μM, the drying temperature was 100 °C, and the final pH of the solution was 4.0 [61].

The FESEM images of GSH incubated Ag colloids at three different concentrations of citrate buffer solution are shown in Fig. 11. At 2.5 mM of citrate buffer solution, the colloids were found to be different sizes and shapes as shown in Fig. 11a. Two typical Ag NPs of diameter 94 and 168 nm have been shown in the inset (i) of Fig. 11a. The insets (i) and (ii) of Fig. 11a represent zoom-in view and corresponding hawk-eye view respectively of a small area (500 nm × 500 nm) as marked by the white square therein. However, at 10 mM of citrate buffer solution, the Ag NPs turned into more uniform along with a typical diameter of 98 nm as shown in Fig. 11b. The insets (i) and (ii) of Fig. 11b display zoom-in view and corresponding hawk-eye view respectively of a small area (500 nm × 500 nm) as marked by the white square therein. However, at higher concentrations of citrate buffer solution such as 10 mM, the particle sizes were found to be increased along with higher aggregation as shown in Fig. 11c. The insets (i) and (ii) of Fig. 11c depict zoom-in view and corresponding hawk-eye view respectively of a small area (500 nm × 500 nm) as marked by the white square therein. It was noted that most of the particles were found uniform along with a higher density of interparticle junctions. Therefore, it was speculated that the GSH incubated Ag NPs in a 10 mM citrate buffer solution should have a considerably larger enhancement factor. The SERS measurements as shown in Fig. 10b indeed verified the speculation.

### 3.6 Role of concentration of Ag NPs on the detection

**Fig. 12:**
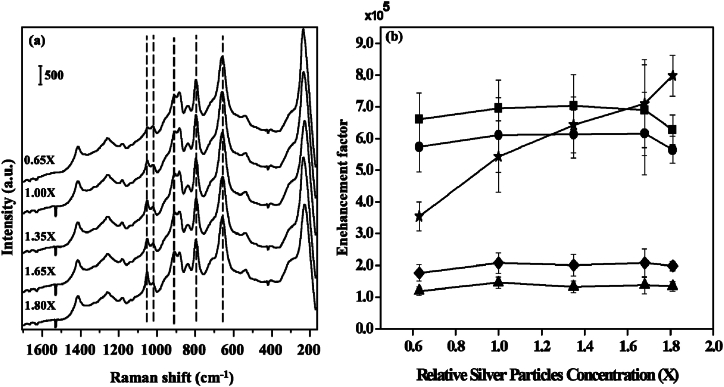
(a) SERS spectra of GSH (10 μM) by heat-induced method for different concentration of Ag colloids. Vertical bar represents the intensity scale. Silver colloids with the reduction time of 15 min were used, the concentration of GSH was 10 μM, the drying temperature was 100 °C, and the final pH of the solution was 4.0 and (b) SERS Enhancement factors of C-S stretching (■), -COO- bending (●), C-C stretching of C-COO- (◆), C-N stretching of glycine terminal (▲), and C-N stretching of glutamic acid terminal (★) calculated from the SERS spectra obtained by heat-induced method for different buffer solution of Ag colloids [61].

The concentration of the participating NPs in fact, increases the site of hotspots as well as the higher possibility of analyte adsorption. To understand the role of the concentration of NPs, SERS spectra of GSH incubated Ag NPs of different concentrations were recorded as shown in Fig. 12a. The enhancement factors were also calculated for different SERS bands under different concentrations of Ag NPs as shown in Fig. 12 b. It is noteworthy to mention that some SERS bands were observed to be enhanced whereas others were mostly unaffected by the concentration of silver particles as shown in Fig. 12b. A detailed in-depth investigation was carried out by FESEM to understand the changes in corresponding morphology at different concentrations of participating Ag NPs.

**Fig. 13:**
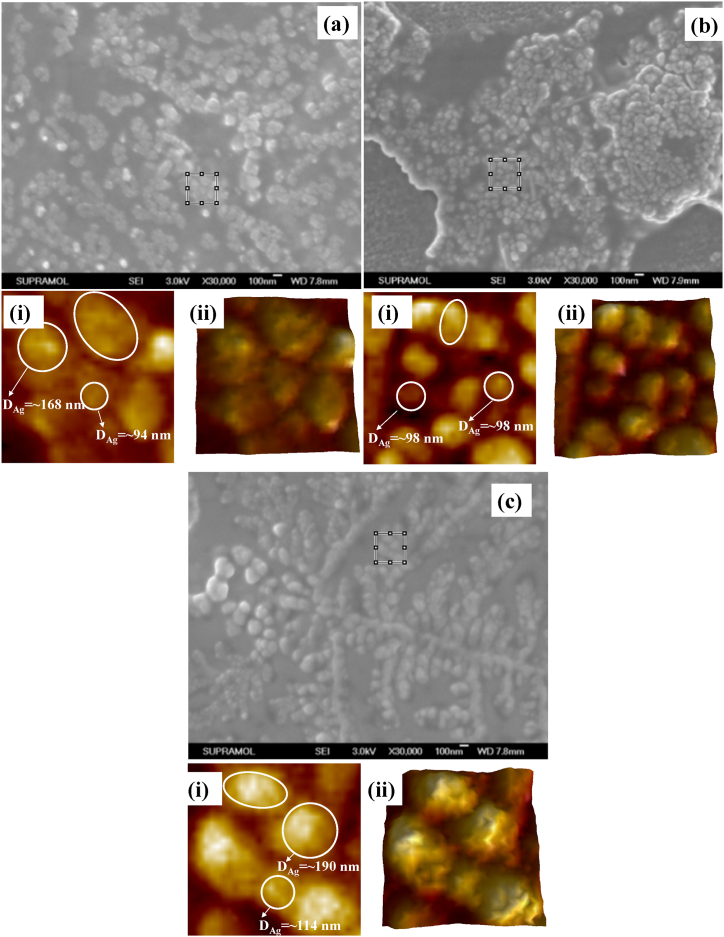
FESEM micrographs of GSH incubated Ag colloids of concentration (a) 0.65 × ; insets (i)-(ii): zoom-in view and corresponding hawk-eye view respectively of a small area (500 nm × 500 nm) as marked by the white square therein, (b) 1.00 × ; insets (i)-(ii): zoom-in view and corresponding hawk-eye view respectively of a small area (500 nm × 500 nm) as marked by the white square therein, and 1.65 × ; insets (i)-(ii): zoom-in view and corresponding hawk-eye view respectively of a small area (500 nm × 500 nm) as marked by the white square therein. The reference Ag NPs with the reduction time of 15 min were used, the GSH concentration was 10 μM, the drying temperature was 100 °C, and the final pH of the solution was 4.0 [61].

Fig. 13 displays the FESEM images of GSH-incubated Ag colloids at three distinct concentrations of Ag NPs. At 0.65 × concentration of participating Ag NPs specimen, the colloids were found to be mostly uniform as shown in Fig. 13a. Two typical Ag NPs of diameter 70 and 82 nm has been shown in the inset (i) of Fig. 13a. The insets (i) and (ii) of Fig. 13a represent zoom-in view and corresponding hawk-eye view respectively of a small area (500 nm × 500 nm) as marked by the white square therein. However, at 1.00 × concentration of participating Ag NPs specimen, the density of Ag NPs was increased along with a typical diameter of 114 nm and 122 nm as shown in Fig. 13b. The insets (i) and (ii) of Fig. 13b provide a magnified view and an overview, respectively, of a tiny region (500 nm × 500 nm) indicated by the white square. However, at higher concentrations of Ag NPs such as 1.65 × concentration of participating Ag NPs specimens, the particle sizes remain mostly the same along with higher aggregation as shown in Fig. 13c. The white square in Fig. 13c indicates a tiny region (500 nm × 500 nm), and insets (i) and (ii) show a zoom-in view and corresponding hawk-eye view of that area, respectively. It was noted that the degree of aggregation and the surface morphologies of the silver aggregation do not show remarkable differences at different concentrations of the participating Ag NPs.

### 3.7 Extension to other amino acids

The suggested heat-induced approach has been compared to existing forms of amino acid detection with reference to GSH detection. Cysteine, methionine, glycine, alanine, valine, serine, threonine, leucine, isoleucine, arginine, lysine, aspartic acid, asparagine, glutamic acid, glutamine, tryptophan, phenylalanine, tyrosine, proline, and histidine were the 20 amino acids for which SERS spectra were detected. The selectivity was examined using the intensity of the 1410 cm^−1^ band caused by the COO-symmetric stretching mode of each acid. Table 2 represents a comparison between the sensitivity of GSH and amino acids. It can be shown that the heat-induced technique exhibits particular selectivity just for GSH and cysteine.

**Table 2:** Heat-induced SERS approach showing SERS intensities of COO-symmetric stretching band at 1410 cm^−1^ for a range of amino acids (10 μM) and GSH.

Samples	SERS intensity of an -COO^-^ symmetric stretching band at 1410 cm^−1^ (a.u.)	Samples	SERS intensity of an -COO^-^ symmetric stretching band at 1410 cm^−1^ (a.u.)
Glutathione	399.2 ± 49.6	Lysine	4.3 ± 2.9
Cysteine	300.2 ± 40.2	Aspartic acid	7.1 ± 4.6
Methionine	26.7 ± 8.6	Asparigine	2.6 ± 2.1
Glycine	6.2 ± 3.9	Glutamic acid	5.6 ± 3.8
Alanine	3.4 ± 3.2	Glutamine	1.5 ± 2.0
Valine	1.55 ± 4.0	Tryptophan	36.9 ± 10.2
Serine	2.0 ± 2.7	Phenylalanine	3.9 ± 2.9
Threonine	2.1 ± 2.1	Tyrosine	2.2 ± 2.4
Leucine	1.7 ± 2.5	Proline	4.8 ± 5.3
Isoleucine	2.2 ± 1.8	Histidine	2.4 ± 4.1
Arginine	2.0 ± 3.2		

## 4 Reversed reporting agent method for glutathione detection

**Fig. 14:**
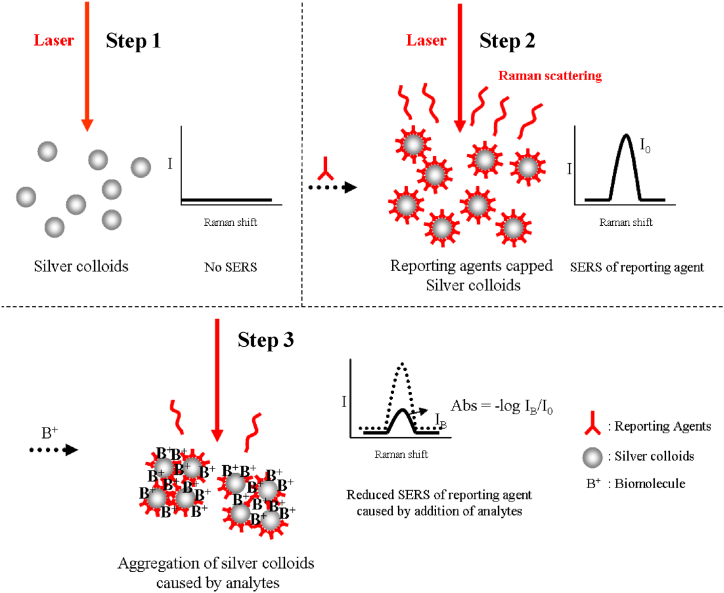
Schematic diagram of reversed reporting agent method for the detection of GSH.

A schematic design of the "reversed reporting agent method" procedure is presented in Fig. 14. Unlike standard Raman-dye labeled processes, Raman-active dye molecules are adsorbed onto the surfaces of Ag colloids as reporting agents, therefore greatly simplifying the labeling process. The morphological alterations in the Ag nanoaggregates impact the SERS signals obtained from the reporting agents during the biomolecule detection process. Furthermore, the rivalry between the analytes at active sites and the reporting agents affects the SERS signals. A quantitative study of the biomolecules was carried out by keeping an eye on the differences in the signals before and after the addition of the sample.

The Ag colloids were fabricated following the procedure mentioned in section 3. A modified Lee and Meisel procedure was employed to create citrate-reduced Ag NP colloidal solutions. In short, an hour was spent aggressively stirring an aqueous solution containing 100 mL of 0.03 percent (w/v) Ag nitrate while it swiftly refluxed to a boiling point. 50 mL of an aqueous sodium citrate solution was added right away. As quickly as possible, the decrease was halted by removing the Ag colloidal solution from the hot plate and placing it in the cold bath. The generated Ag colloidal solution was mixed with 150 mL of citrate buffer solution (10 mM and pH of 4) prior to the addition of the reporting agent solution. The colloids of Ag NPs were further stabilized as a result. The high concentration (1 mM) water solution of the selected reporting agent was combined with the Ag colloidal solution to pre-equilibrate it before the GSH detection. The final concentration of reporting agent in the substrate solution was therefore 10 μM after pre-equilibrium. The sample solution is made in the same way as the reporting agent capped Ag colloidal solution by spiking 10 μL of concentrated GSH solution into 1 mL of the substrate solution.

### 4.1 Selection of reporting agents

**Fig. 15:**
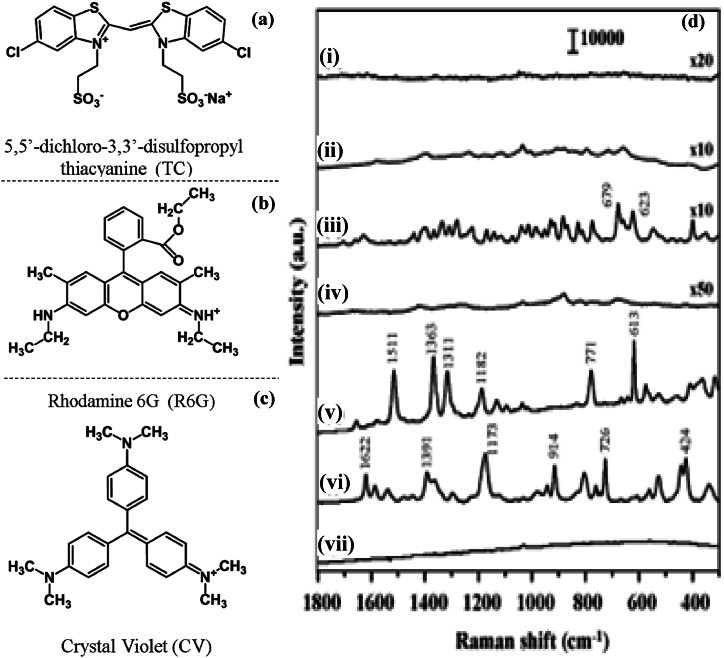
(a) Molecular structures of three reporting agents; top: 5,5′-dichloro-3,3′-disulfopropyl thiacyanine (TC), middle: Rhodamine 6G (R6G) and below: crystal violet (CV)) used in reversed reporting agent method. (b) SERS and Raman spectra; inset (i)-(iii) 500 μM GSH incubated Ag colloid, Raman spectra of GSH powder, and Raman spectra of 0.5 M GSH aqueous solution as reference respectively, and inset (iv)-(vi): SERS spectra of 5 μM R6G, 5 μM of CV and 5 μM of TC respectively (vi). The pH of sample solutions was 4.0 and the Raman exposure times were 30 s with 1 accumulation for (i)-(iii) and 1 s with 10 accumulations for (iv)-(vii). Vertical bar represents the intensity scale [63].

The SERS spectra of 500 μM GSH recorded from the NaBH_4_^−^ and citrate-reduced Ag colloids, respectively, are displayed in the insets (i)-(ii) of Fig. 15 respectively. For comparison, the standard Raman spectra of GSH powder and that of a concentrated 0.5 M GSH aqueous solution were shown in the insets (iii)-(iv) of Fig. 15 respectively. Fig. 15i revealed that using NaBH_4_-reduced Ag colloids as the SERS substrate resulted in no notable SERS signal of GSH. However, using the reversed reporting agent approach, the enhancement factor in SERS for GSH detection was improved. Prior to GSH detection, the surfaces of Ag colloids were capped with three distinct dye molecules exhibiting intense resonance Raman effect: rhodamine 6G (C_28_H_31_N_2_O_3_Cl, R6G), crystal violet (C_25_H_30_N_3_Cl, CV), and 5,5′-dichloro-3,3′-disulfopropyl thiacyanine (C_19_H_15_C_l2_N_2_NaO_6_S_4_, TC) as shown in Fig. 15a-c. The citrate-reduced Ag colloids containing the three reporting agents under investigation have their SERS spectra displayed in the insets (v)-(vi) of Fig. 15 respectively. While a SERS spectrum of TC cannot be produced, band locations of observed R6G and CV SERS spectra are in good agreement with those reported so far. It was deduced that R6G and CV, which were identified by citrate-reduced Ag colloids, had the ability to function as reporting agents.

### 4.2 Selectivity to glutathione detection

**Fig. 16:**
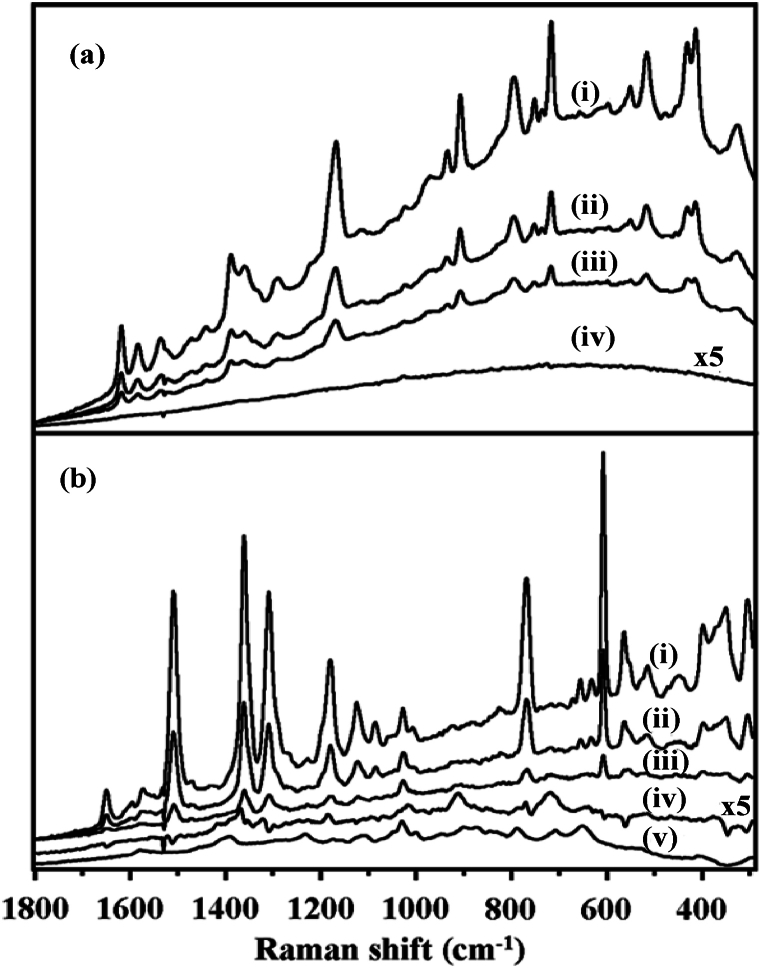
(a) The SERS spectra of CV and GSH; insets (i)-(iii): 5 μM CV, after addition of 10 μM GSH, and 100 μM GSH respectively and inset (iv): The removal of CV before and after the addition of 100 μM GSH and (b) The SERS spectra of R6G and GSH; insets (i)-(iii): 5 μM R6G, after addition of 10 μM GSH, and 100 μM GSH respectively, inset (iv): The removal of R6G before and after the addition of 100 μM GSH and inset (v): the SERS spectrum of 500 μM GSH detected with citrate-reduced Ag colloids as reference [63].

According to preliminary screening, the detection of GSH was confirmed by pre-adjusting R6G and CV with GSH incubated Ag colloids. SERS spectra of 5 μM CV pre-equilibrated Ag colloids and those following the addition of 10 μM and 100 μM GSH solution are shown in the insets (i)-(iii) of Fig. 16a respectively. The subtraction of CV before and after the addition of 100 μM GSH solution was shown in the inset (iv) of Fig. 16a. The comparable SERS spectra in the case of 5 μM R6G are shown in the insets (i)-(iii) of Fig. 16b. It is shown that the addition of GSH results in a reduction of the SERS signals of reporting agents, and that the degree of this reduction is correlated with the concentrations of GSH solutions analyzed. It is feasible to infer two explanations for this observation. The first is the rivalry between GSH molecules and reporting agents on the Ag surfaces, which results in the additional aggregation of Ag colloids due to GSH. It is commonly acknowledged that the morphology of a substrate has a significant impact on the SERS intensity as explained in Fig. 6, Fig. 13. GSH addition would likely lead to further Ag colloidal aggregation; therefore, the Ag aggregate shape would no longer be appropriate for triggering strong SERS. On the other hand, GSH may take the place of the reporting agents immobilized on the Ag surfaces because of the remarkably strong interaction between a thiol functional group and Ag. In order to validate this hypothesis, the SERS spectra of the reporting agents were subtracted from the SERS spectra of the agents prior to the addition of 100 μM GSH. The resulting subtracted data are displayed in the inset (iv) of Fig. 16a and in the inset (iv) of Fig. 16b. The subtracted spectrum does not indicate the presence of GSH adsorbed on the Ag surfaces when CV was utilized as the reporting agent. Conversely, when R6G was used as the reporting agent, the subtraction result showed evidence of GSH adsorbed on the surfaces of Ag colloids. Raman peaks at 650, 725, 793, 913, and 1020 cm^−1^ were seen, as shown in inset (iv) of Fig. 16b. These peaks could be attributed to the C-S stretching, -COO-deformation, -COO-bending, C-COO-stretching, and C-N stretching of GSH. For simplicity of comparison, the SERS spectra of 500 μM GSH identified using the same Ag colloids have been shown in the inset (v) of Fig. 16b. This discovery shows that the reversed reporting agent strategy suggested in this work may preserve the spectrum information about analytes. Furthermore, band shifts of R6G were seen with the addition of GSH, indicating that either the morphology of Ag colloids was altered or that GSH and the reporting agent interacted. However, as for CV, no such outcome was noted.

## 5 Perspective, outlook and conclusion

As mentioned early, GSH plays a crucial role in maintaining cellular health and protecting our bodies from oxidative damage. Therefore, the detection of GSH has attracted significant attention in the field of oncology. The perspective on GSH detection is multifaceted, encompassing both the scientific advancements made in detection techniques and the potential impact on human health [64,65]. From a scientific standpoint, the development of accurate and sensitive methods for detecting GSH has been a priority. GSH levels in biological samples have been measured using a variety of analytical methods, including mass spectrometry, enzymatic tests, and HPLC. With the use of these methods, practitioners can examine the dynamics of GSH metabolism, track variations in its concentration under various circumstances, and look into the function it plays in a number of disorders [66,67].

The future of GSH detection is bright, as new techniques and applications continue to be developed thanks to continuous technological and scientific breakthroughs. The potential for detection approaches to transform diagnostics and therapies is becoming more and more apparent as scientists continue to explore the complex mechanisms of GSH and its function in both health and sickness [66]. The development of non-invasive or minimally invasive detection techniques is one area of interesting advancement [68]. Conventional methods sometimes call for intrusive procedures, such as blood samples, which can cause patients discomfort and reduce the number of tests that can be performed. But new developments in sensor technology, such as wearable biosensors, make it possible to track GSH levels continuously and in real time [69]. This would give medical practitioners important knowledge regarding the dynamic fluctuations in GSH concentration, enabling more individualized treatment regimens and better disease control. Furthermore, there is a lot of potential for GSH detection to be integrated into point-of-care equipment [70]. Customized and user-friendly diagnostic instruments that can quickly assess GSH levels at onsite or patients in environments with limited resources will significantly improve the effectiveness and accessibility of healthcare [71,72]. These gadgets have the potential to completely transform illness detection, tracking, and treatment response evaluation, especially in underdeveloped or isolated places with inadequate laboratory facilities. An intriguing direction in the search for GSH detection is the investigation of new biomarkers and imaging methods. Given the involvement of GSH in oxidative stress and redox signaling, measuring it might be a useful way to monitor the health of cells and the course of illness. The potential of biomarkers and imaging agents that can precisely target and visualize GSH in tissues is being intensively investigated by researchers. These developments may help with early diagnosis, direct treatment measures, and offer insightful information on the processes underlying illness. GSH detection may be revolutionized as a result of the use of machine learning techniques and artificial intelligence (AI) [73]. Artificial intelligence (AI) algorithms can help in the creation of prediction models for illness diagnosis and prognosis by examining massive datasets and finding trends. This might result in more precise and customized treatment regimens, improving the outcomes for patients.

Finally, it should be noted that the area of GSH detection has advanced significantly in recent years and offers bright futures for a variety of uses. A greater comprehension of the function of GSH in health and illness has been made possible by the development of sensitive and reliable detection techniques. GSH detection has the enormous potential to transform several facets of healthcare, from scientific research to clinical diagnosis and therapeutic treatments. Technological developments like wearables and biosensors make it possible to continuously and non-invasively measure GSH levels. This allows for more effective disease management and individualized treatment approaches by providing healthcare practitioners with insightful information about the dynamic fluctuations in GSH concentration. The role of GSH in oxidative stress and redox signaling has been clarified by the investigation of new biomarkers and imaging modalities. These developments may help with early diagnosis, direct treatment measures, and offer insightful information on the processes underlying illness. GSH detection is seen from a variety of angles outside of medicine. Monitoring GSH levels in people exposed to pollutants or toxins can be beneficial for research on occupational and environmental health, since it can help us understand how the body defends itself and how environmental variables affect health. GSH metabolism is a complicated process, and further study will surely reveal more details. Nevertheless, identifying this process is essential to improving health. Future developments in GSH detection are quite promising, provided that scientists, physicians, and technology developers work together to make more progress. Personalized treatment, early intervention, and improved patient outcomes will all be made possible by the capacity to quantify GSH levels correctly in real-time with enhanced sensitivity and specificity. In the end, GSH detection has the potential to revolutionize healthcare by offering insightful knowledge about the state of cells, the course of diseases, and the effectiveness of treatments. This will eventually improve the quality of life for individuals around the world.

## CRediT authorship contribution statement

**Mohammad Kamal Hossain:** Writing – review & editing, Writing – original draft, Resources, Funding acquisition, Formal analysis, Conceptualization. **Genin Gary Huang:** Writing – review & editing, Investigation, Formal analysis, Conceptualization. **Mohammad Mozahar Hossain:** Writing – review & editing, Supervision, Funding acquisition.

## Declaration of competing interest

The authors declare that they have no known competing financial interests or personal relationships that could have appeared to influence the work reported in this paper.

## References

[1] Santra T.S., Tseng F.G. (2023). Single Biomolecule Detection and Analysis: Concepts, Applications, and Future Prospects.

[2] Mohankumar P., Ajayan J., Mohanraj T., Yasodharan R. (2021). Recent developments in biosensors for healthcare and biomedical applications: a review. Measurement.

[3] Gupta U., Gupta V., Arun R.K., Chanda N. (2022). Recent advances in enzymatic biosensors for point‐of‐care detection of biomolecules. Biotechnol. Bioeng..

[4] Paramasivam G., Sanmugam A., Palem V.V., Sevanan M., Sairam A.B., Nachiappan N., Youn B., Lee J.S., Nallal M., Park K.H. (2024). Nanomaterials for detection of biomolecules and delivering therapeutic agents in theragnosis: a review. Int. J. Biol. Macromol..

[5] Su Z., Li T., Wu D., Wu Y., Li G. (2022). Recent progress on single-molecule detection technologies for food safety. J. Agric. Food Chem..

[6] Sheng K., Jiang H., Fang Y., Wang L., Jiang D. (2022). Emerging electrochemical biosensing approaches for detection of allergen in food samples: a review. Trends Food Sci. Technol..

[7] Wan N., Shi J., Xu J., Huang J., Gan D., Tang M., Li X., Huang Y., Li P. (2023). Gasdermin D: a potential new auxiliary pan-biomarker for the detection and diagnosis of diseases. Biomolecules.

[8] Paramasivam G., Sanmugam A., Palem V.V., Sevanan M., Sairam A.B., Nachiappan N., Youn B., Lee J.S., Nallal M., Park K.H. (2024). Nanomaterials for detection of biomolecules and delivering therapeutic agents in theragnosis: a review. Int. J. Biol. Macromol..

[9] Iqbal M.J., Javed Z., Herrera-Bravo J., Sadia H., Anum F., Raza S., Tahir A., Shahwani M.N., Sharifi-Rad J., Calina D., Cho W.C. (2022). Biosensing chips for cancer diagnosis and treatment: a new wave towards clinical innovation. Cancer Cell Int..

[10] Bagatini M., Assmann C., Blumenberg M. (2020).

[11] Adeoye O., Olawumi J., Opeyemi A., Christiania O. (2018). Review on the role of glutathione on oxidative stress and infertility. JBRA assisted reproduction.

[12] Matuz-Mares D., Riveros-Rosas H., Vilchis-Landeros M.M., Vázquez-Meza H. (2021). Glutathione participation in the prevention of cardiovascular diseases. Antioxidants.

[13] Raj Rai S., Bhattacharyya C., Sarkar A., Chakraborty S., Sircar E., Dutta S., Sengupta R. (2021). Glutathione: role in oxidative/nitrosative stress, antioxidant defense, and treatments. ChemistrySelect.

[14] Kumar A., Dhull D.K., Gupta V., Channana P., Singh A., Bhardwaj M., Ruhal P., Mittal R. (2017). Role of Glutathione-S-transferases in neurological problems. Expert Opin. Ther. Pat..

[15] Mazari A.M., Zhang L., Ye Z.W., Zhang J., Tew K.D., Townsend D.M. (2023). The multifaceted role of glutathione s-transferases in health and disease. Biomolecules.

[16] Yin G., Gan Y., Jiang H., Yu T., Liu M., Zhang Y., Li H., Yin P., Yao S. (2021). Direct quantification and visualization of homocysteine, cysteine, and glutathione in Alzheimer's and Parkinson's disease model tissues. Anal. Chem..

[17] Aborode A.T., Pustake M., Awuah W.A., Alwerdani M., Shah P., Yarlagadda R., Ahmad S., Silva Correia I.F., Chandra A., Nansubuga E.P., Abdul-Rahman T. (2022). Targeting oxidative stress mechanisms to treat Alzheimer's and Parkinson's disease: a critical review. Oxid. Med. Cell. Longev..

[18] Iskusnykh I.Y., Zakharova A.A., Pathak D. (2022). Glutathione in brain disorders and aging. Molecules.

[19] Hossain M.K., Ozaki Y. (2009). Surface-enhanced Raman scattering: facts and inline trends. Curr. Sci..

[20] Hossain M.K. (2013, July).

[21] Mallik D.C.V. (2000). The Raman effect and Krishnan's diary. Notes and Records of the Royal Society of London.

[22] Suhito I.R., Koo K.M., Kim T.H. (2020). Recent advances in electrochemical sensors for the detection of biomolecules and whole cells. Biomedicines.

[23] Denoroy L., Zimmer L., Renaud B., Parrot S. (2013). Ultra high performance liquid chromatography as a tool for the discovery and the analysis of biomarkers of diseases: a review. J. Chromatogr. B.

[24] Zhang J., Wang X.Y., Wang Y.H., Wang D.D., Song Z., Zhang C.D., Wang H.S. (2020). Colorable zeolitic imidazolate frameworks for colorimetric detection of biomolecules. Anal. Chem..

[25] Hu J., Yan X., Chris Le X. (2024). Label-free detection of biomolecules using inductively coupled plasma mass spectrometry (ICP-MS). Anal. Bioanal. Chem..

[26] Kumar S., Singh R. (2021). Recent optical sensing technologies for the detection of various biomolecules. Opt Laser. Technol..

[27] Cid-Barrio L., Calderón-Celis F., Costa-Fernández J.M., Encinar J.R. (2020). Assessment of the potential and limitations of elemental mass spectrometry in life sciences for absolute quantification of biomolecules using generic standards. Anal. Chem..

[28] Steullet P., Cabungcal J.H., Kulak A., Cuenod M., Schenk F., Do K.Q. (2011). Glutathione deficit and redox dysregulation in animal models of schizophrenia. Animal Models of Schizophrenia and Related Disorders.

[29] Hu J., Liu F., Chen Y., Shangguan G., Ju H. (2021). Mass spectrometric biosensing: a powerful approach for multiplexed analysis of clinical biomolecules. ACS Sens..

[30] Li C.C., Li Y., Zhang Y., Zhang C.Y. (2020). Single-molecule fluorescence resonance energy transfer and its biomedical applications. TrAC, Trends Anal. Chem..

[31] Beeram R., Vepa K.R., Soma V.R. (2023). Recent trends in SERS-based plasmonic sensors for disease diagnostics, biomolecules detection, and machine learning techniques. Biosensors.

[32] Banchelli M., Amicucci C., Ruggiero E., D'Andrea C., Cottat M., Ciofini D., Osticioli I., Ghini G., Siano S., Pini R., de Angelis M. (2019). Spot‐on SERS detection of biomolecules with laser‐patterned dot arrays of assembled silver nanowires. ChemNanoMat.

[33] Juang R.S., Wang K.S., Cheng Y.W., Fu C.C., Chen W.T., Liu C.M., Chien C.C., Jeng R.J., Chen C.C., Liu T.Y. (2019). Floating SERS substrates of silver nanoparticles-graphene based nanosheets for rapid detection of biomolecules and clinical uremic toxins. Colloids Surf. A Physicochem. Eng. Asp..

[34] Lee S., Kwon S., Lee S., Oh M.J., Jung I., Park S. (2024). Combinatorial effect of tricomponent dual-rim nanoring building blocks: label-free SERS detection of biomolecules. Nano Lett.

[35] Jebakumari K.E., Murugasenapathi N.K., Palanisamy T. (2023). Engineered two-dimensional nanostructures as SERS substrates for biomolecule sensing: a review. Biosensors.

[36] Larsson M., Lindgren J. (2005). Analysis of glutathione and immunoglobulin G inside chromatographic beads using surface‐enhanced Raman scattering spectroscopy. J. Raman Spectrosc.: An International Journal for Original Work in all Aspects of Raman Spectroscopy, Including Higher Order Processes, and also Brillouin and Rayleigh Scattering.

[37] Huang G.G., Han X.X., Hossain M.K., Ozaki Y. (2009). Development of a heat-induced surface-enhanced Raman scattering sensing method for rapid detection of glutathione in aqueous solutions. Anal. Chem..

[38] Altun A.O., Bond T., Pronk W., Park H.G. (2017). Sensitive detection of competitive molecular adsorption by surface-enhanced Raman spectroscopy. Langmuir.

[39] Kitahama Y., Hossain M.K., Ozaki Y., Itoh T., Sujith A., Han X. (2010). Surface‐enhanced Raman scattering imaging: application and experimental approach by far‐field with conventional setup. Raman, Infrared, and Near‐Infrared Chemical Imaging.

[40] Kamal Hossain M. (2022). Nanoscale imaging of interstitial‐dependent optical confinement through near‐field scanning optical microscopy. Chem. Rec..

[41] Hossain M.K., Kitajima M., Imura K., Okamoto H. (2017). Interstitial-dependent enhanced photoluminescence: a near-field microscopy on single spheroid to dimer, tetramer, and few particles gold nanoassembly. J. Phys. Chem. C.

[42] Schatz G.C. (2021). SERS and the scientific career of Richard P. Van Duyne (1945–2019). J. Raman Spectrosc..

[43] Creighton J.A. (2010). Contributions to the early development of surface-enhanced Raman spectroscopy. Notes Record Roy. Soc. Lond..

[44] Graham D., Moskovits M., Tian Z.Q. (2017). SERS–facts, figures and the future. Chem. Soc. Rev..

[45] Park W.H., Kim Z.H. (2010). Charge transfer enhancement in the SERS of a single molecule. Nano Lett..

[46] Langer J., Jimenez de Aberasturi D., Aizpurua J., Alvarez-Puebla R.A., Auguié B., Baumberg J.J., Bazan G.C., Bell S.E., Boisen A., Brolo A.G., Choo J. (2019). Present and future of surface-enhanced Raman scattering. ACS Nano.

[47] Albrecht M.G., Creighton J.A. (1977). Anomalously intense Raman spectra of pyridine at a silver electrode. J. Am. Chem. Soc..

[48] Yu Y., Xiao T.H., Wu Y., Li W., Zeng Q.G., Long L., Li Z.Y. (2020). Roadmap for single-molecule surface-enhanced Raman spectroscopy. Advanced Photonics.

[49] Qiu Y., Kuang C., Liu X., Tang L. (2022). Single-molecule surface-enhanced Raman spectroscopy. Sensors.

[50] Choi H.K., Lee K.S., Shin H.H., Koo J.J., Yeon G.J., Kim Z.H. (2019). Single-molecule surface-enhanced Raman scattering as a probe of single-molecule surface reactions: promises and current challenges. Accounts Chem. Res..

[51] Sinha R.K. (2023). Surface-enhanced Raman scattering and localized surface plasmon resonance detection of aldehydes using 4-ATP functionalized Ag nanorods. Plasmonics.

[52] Nanda B.P., Rani P., Paul P., Bhatia R. (2024). Recent trends and impact of localized surface plasmon resonance (LSPR) and surface-enhanced Raman spectroscopy (SERS) in modern analysis. Journal of Pharmaceutical Analysis.

[53] Hossain M.K. (2022). Silver-decorated silicon nanostructures: fabrication and characterization of nanoscale terraces as an efficient SERS-active substrate. Int. J. Mol. Sci..

[54] Hossain M.K., Drmosh Q.A., Arifuzzaman M. (2022). Silver nanoparticles, nanoneedles and nanorings: impact of electromagnetic near-field on surface-enhanced Raman scattering. Phys. Chem. Chem. Phys..

[55] Hossain M.K., Drmosh Q.A., Mohamedkhair A.K. (2021). Plasmonic pollen grain nanostructures: a three‐dimensional surface‐enhanced Raman scattering (SERS)‐Active substrate. Chem.--Asian J..

[56] Hossain M.K., Mukhaimer A.W., Al-Jabari M. (2021). Fabrication and characterizations of arbitrary-shaped silver nanoparticles for surface-enhanced fluorescence microscopy. J. Nanoparticle Res..

[57] Hossain M.K., Kitahama Y., Ozaki Y. (2021). Half-raspberry-like bimetallic nanoassembly: interstitial dependent correlated surface plasmon resonances and surface-enhanced Raman spectroscopy. Phys. Chem. Chem. Phys..

[58] Hossain M.K. (2020). Nanoassembly of gold nanoparticles: an active substrate for size-dependent surface-enhanced Raman scattering. Spectrochim. Acta Mol. Biomol. Spectrosc..

[59] Haruna K., Saleh T.A., Hossain M.K., Al-Saadi A.A. (2016). Hydroxylamine reduced silver colloid for naphthalene and phenanthrene detection using surface-enhanced Raman spectroscopy. Chem. Eng. J..

[60] Hossain M.K., Huang G.G., Tanaka Y., Kaneko T., Ozaki Y. (2015). Anisotropic gold nanoassembly: a study on polarization-dependent and polarization-selective surface-enhanced Raman scattering. Phys. Chem. Chem. Phys..

[61] Huang G.G., Han X.X., Hossain M.K., Kitahama Y., Ozaki Y. (2010). A study of glutathione molecules adsorbed on silver surfaces under different chemical environments by surface-enhanced Raman scattering in combination with the heat-induced sensing method. Appl. Spectrosc..

[62] Larsson M., Lindgren J. (2005). Analysis of glutathione and immunoglobulin G inside chromatographic beads using surface‐enhanced Raman scattering spectroscopy. J. Raman Spectrosc.: An International Journal for Original Work in all Aspects of Raman Spectroscopy, Including Higher Order Processes, and also Brillouin and Rayleigh Scattering.

[63] Huang G.G., Hossain M.K., Han X.X., Ozaki Y. (2009). A novel reversed reporting agent method for surface-enhanced Raman scattering; highly sensitive detection of glutathione in aqueous solutions. Analyst.

[64] Wang Z., Xu K.F., Wang G., Durrani S., Lin F., Wu F.G. (2023). “One stone, five birds”: ultrabright and multifaceted carbon dots for precise cell imaging and glutathione detection. Chem. Eng. J..

[65] Zhao J., Li X., Ma T., Chang B., Zhang B., Fang J. (2023). Glutathione‐triggered prodrugs: design strategies, potential applications, and perspectives. Med. Res. Rev..

[66] Rattanawong K., Koiso N., Toda E., Kinoshita A., Tanaka M., Tsuji H., Okamoto T. (2021). Regulatory functions of ROS dynamics via glutathione metabolism and glutathione peroxidase activity in developing rice zygote. Plant J..

[67] Timme-Laragy A.R., Goldstone J.V., Imhoff B.R., Stegeman J.J., Hahn M.E., Hansen J.M. (2013). Glutathione redox dynamics and expression of glutathione-related genes in the developing embryo. Free Radic. Biol. Med..

[68] Mandal P.K., Tripathi M., Sugunan S. (2012). Brain oxidative stress: detection and mapping of anti-oxidant marker ‘Glutathione’in different brain regions of healthy male/female, MCI and Alzheimer patients using non-invasive magnetic resonance spectroscopy. Biochemical and biophysical research communications.

[69] Yan T., Zhang G., Chai H., Qu L., Zhang X. (2021). Flexible biosensors based on colorimetry, fluorescence, and electrochemistry for point-of-care testing. Front. Bioeng. Biotechnol..

[70] Yuan X., Bai F., Ye H., Zhao H., Zhao L., Xiong Z. (2021). Smartphone-assisted ratiometric fluorescence sensing platform and logical device based on polydopamine nanoparticles and carbonized polymer dots for visual and point-of-care testing of glutathione. Anal. Chim. Acta.

[71] Tomei M.R., Cinti S., Interino N., Manovella V., Moscone D., Arduini F. (2019). Based electroanalytical strip for user-friendly blood glutathione detection. Sensor. Actuator. B Chem..

[72] Yuan K., Cuntín-Abal C., Jurado-Sánchez B., Escarpa A. (2021). Smartphone-based Janus micromotors strategy for motion-based detection of glutathione. Anal. Chem..

[73] Pantic I., Paunovic J., Pejic S., Drakulic D., Todorovic A., Stankovic S., Vucevic D., Cumic J., Radosavljevic T. (2022). Artificial intelligence approaches to the biochemistry of oxidative stress: current state of the art. Chem. Biol. Interact..

